# A Cross-Sectional Study to Determine the Association of Corrected QT Interval With Microalbuminuria in Type 2 Diabetes Mellitus

**DOI:** 10.7759/cureus.38967

**Published:** 2023-05-13

**Authors:** Sonali Sawarthia, Rishi Patel, Prajakta P Patil

**Affiliations:** 1 Internal Medicine, Bharati Vidyapeeth Medical College and Hospital, Pune, IND

**Keywords:** cardiac autonomic neuropathy, high blood glucose levels, microalbuminuria (ma), diabetes type 2, qt interval prolongation

## Abstract

Introduction

Cardiac autonomic neuropathy (CAN) is a frequent and life-threatening complication of type 2 diabetes. Failure to diagnose can lead to high mortality and morbidity. In patients who have diabetes mellitus, microalbuminuria is an independent marker for cardiovascular disease. This study aimed to assess the corrected QT interval with microalbuminuria in type 2 diabetes mellitus. The objective of this study was to estimate the corrected QT interval in subjects with type 2 diabetes mellitus and to determine the association of the corrected QT interval with microalbuminuria type 2 diabetes mellitus.

Methodology

Ninety-five adult patients (>18 years to 65 years) diagnosed with type 2 diabetes mellitus with microalbuminuria were included in this study. Data were collected on the proforma through history taking and a general physical and systemic examination. An electrocardiograph was taken on the day of admission; the most prolonged QT interval was measured, and the RR interval was calculated. The data were statistically analyzed using IBM SPSS Statistics for Windows, Version 24 (Released 2016; IBM Corp., Armonk, New York, United States).

Results

There was a significant difference in the corrected QT interval prolongation prevalence between diabetic patients with microalbuminuria and without microalbuminuria (P-value <0.001). The mean corrected QT interval distribution did not differ significantly across various age groups of cases studied with microalbuminuria (P-value 0.98). The distribution of mean corrected QT interval did not differ significantly between the group of male cases and group of female cases studied with microalbuminuria (P-value 0.66). The mean corrected QT interval distribution did not differ significantly across various duration of diabetes groups among the cases studied with microalbuminuria (P-value 0.60). The mean corrected QT interval distribution did not differ significantly across different types of anti-diabetic treatment groups among the cases studied with microalbuminuria (P-value 0.64).

Conclusion

Type 2 diabetes has been prevalent in Indian and Asian populations. The early management of type 2 diabetes is necessary since the early stages of the disease can reduce the risk of CAN. Therefore, these patients should be diagnosed as early as possible and treated to reduce associated mortality and risk and to improve quality of care.

## Introduction

Diabetes belongs to a group of common metabolic disorders that leads to hyperglycemia [1]. It is estimated that around 171 million people in the world have diabetes mellitus in the year 2000, and it is projected to increase to 366 million by 2030 [1]. Type 2 diabetes mellitus is expected in the age group >30 years. These subjects have a further increased risk for macrovascular complications like ischemic heart disease and peripheral vascular diseases, including sudden cardiac death [2,3]. Each of the microvascular complications is found to be an independent risk factor for sudden cardiac death in diabetes [4].

In 1974, Parving et al. ﬁrst described microalbuminuria (an abnormal increase in the urinary excretion rate of albumin to between 30 and 300mg/24h) in patients with diabetes mellitus [2]. Parving et al. also demonstrated that microalbuminuria was associated with essential hypertension in non-diabetic subjects. In the early 1980s, several longitudinal studies in type I and type II diabetes documented that microalbuminuria was an influential independent risk factor for diabetic nephropathy [3,5]. Later, several prospective epidemiologic studies demonstrated that microalbuminuria is a critical risk factor for cardiovascular disease in type II diabetic patients. A meta-analysis of these studies showed that the presence of microalbuminuria doubles the risk of cardiovascular morbidity or mortality [6]. Observational studies in non-diabetic subjects have revealed that microalbuminuria is an independent, decisive risk factor for fatal and non-fatal vascular events and all-cause mortality [7,8].

The QT interval in an electrocardiogram (ECG) indicates the time taken for ventricular depolarization and repolarization [2]. Its correction with heart rate is called the corrected QT interval. Due to the non-uniform repolarization of the myocardium, the corrected QT interval is prolonged in diabetes, leading to a lowered threshold for ventricular fibrillation and torsades de pointes [9,10]. Studies have shown that prolonged corrected QT intervals are predictors of cardiovascular mortality [11-13].

Ninkovic et al. showed that 44.1% of the study population with type 2 diabetes mellitus has prolonged corrected QT intervals [9]. Therefore, the present study is undertaken to associate the corrected QT interval with the microvascular complications in subjects with type 2 diabetes as it is a cost-effective and simple method to predict cardiovascular risk so that early detection and prevention will reduce mortality and morbidity.

This study aimed to evaluate the corrected QT interval with microalbuminuria in type 2 diabetes mellitus. The objectives of this study were to estimate corrected QT interval in subjects with type 2 diabetes mellitus and to determine the association of the corrected QT interval with microalbuminuria type 2 diabetes mellitus.

## Materials and methods

This prospective cross-sectional study was done at Bharati Vidyapeeth Hospital, a tertiary healthcare center, in Pune, India, from January 2021 to June 2022. The Institutional Ethics Committee approval was taken, and written informed consent was obtained from all the cases included in this study. The study was conducted in accordance with Helsinki declaration. The sample size was estimated to be 90 by an expert statistician using previous literature effect size. 

Ninety-five adult patients (>18 years to 65 years) diagnosed with type 2 diabetes mellitus with microalbuminuria were included in this study. Subjects over 65 years were excluded as they are at a higher risk for cardiac autonomic neuropathy (CAN).

Patients with a history of ischemic heart disease, myocarditis, cerebrovascular stroke, subarachnoid hemorrhage, encephalitis, head injury, pacemakers, hypothyroidism, electrolyte imbalance, atrial ﬁbrillation, major ventricular conduction defects (deﬁned as long QRS duration >120 ms), and left ventricular hypertrophy were excluded from this study. Patients who were on medications that affect the QT interval, such as anti-arrhythmic agents (amiodarone, quinidine, disopyramide), anti-psychotic agents (haloperidol, droperidol, ziprasidone), and anti-depressants (amitriptyline, desipramine, sertraline), digoxin, ketoconazole, and fluoroquinolones (levofloxacin, ciprofloxacin), were excluded in this study. Patients with a history of alcohol intake or gestational diabetes were also excluded from this study.

All the participants were screened for the presence of type 2 diabetes. Subjects fulfilling the inclusion criteria were enrolled in the study. All the subjects from the department of general medicine and endocrinology were included. Data were collected as per the predecided through history taking, including name, age, sex, history of diabetes, treatment history, and drug history, as well as through a general physical examination with height, weight, BMI, pulse rate, and blood pressure, and systemic examination was done. An electrocardiograph was taken on the day of admission; the most prolonged QT interval was measured manually. It was measured from the start of the QRS complex to the end of the downslope of the T wave (crossing the isoelectric line) in the lead II. The RR interval was calculated by manually counting the number of small boxes between the two peaks of the R wave in lead II.

Assessment measures

QT and RR intervals were measured manually.

Corrected QT interval (QT c) by Bazett 1920 et al. [14].

QT c = QT/ √RR interval

Type 2 diabetes mellitus as per criteria laid by the American Diabetes Association 2018, any one of the below criteria: Fasting blood glucose ≥126 mg/dL, postprandial blood sugar ≥200 mg/dL, HbA1c ≥6.5 gm%, and random blood sugar ≥200mg/dL with osmotic symptoms (polyuria, polydipsia, weight loss) [14].

Nephropathy was diagnosed with the help of urine albumin creatinine ratio. Creatinine level <30 mg/g creatinine was considered normal, and creatinine level 30-300 mg/g creatinine was considered microalbuminuria. Creatinine level >300 mg/g was considered proteinuria. Typically, the QTc value is ≤0.44 seconds. It is prolonged if > 0.45 sec in males and >0.46 sec in females [15].

Statistical data analysis

The data are statistically analyzed using IBM SPSS Statistics for Windows, Version 24 (Released 2016; IBM Corp., Armonk, New York, United States) for MS Windows. The data on categorical variables are shown as n (% of cases), and the data on continuous variables are presented as mean and standard deviation (SD). The inter-group statistical comparison of the distribution of categorical variables is tested using the Chi-Square test or Fisher's exact probability test if more than 20% of cells have an expected frequency of less than 5. All results are shown in a tabular and graphical format to visualize the statistically significant difference. In the study, the P-values less than 0.05 are considered statistically significant.

## Results

We studied 95 cases with microalbuminuria and 180 cases without microalbuminuria. Of 95 cases with microalbuminuria, 16 cases (16.8%) had an age between 30 and 39 years, 17 cases (17.9%) had an age between 40 and 49 years, 27 cases (28.4%) had an age between 50 and 59 years, and the majority of cases (36.8%) had an age between 60 and 65 years. The mean ± SD of the age of cases with microalbuminuria was 52.6 ± 10.2 years, and the minimum - maximum age range was 30 - 65 years.

The age distribution of cases studied differs significantly between the group of cases with microalbuminuria and those without microalbuminuria (P-value<0.05). Of 95 cases studied with microalbuminuria, 51 cases (53.7%) were male, and 44 (46.3%) were female. The male-to-female sex ratio was 1.16: 1.00 in the study group. The sex distribution of cases studied differs significantly between a group of cases with microalbuminuria (n=95) and a group of cases without microalbuminuria (n=180) (P-value<0.046).

Of 95 cases studied with microalbuminuria, two cases (2.1%) had BMI less than 18.50 kg/m^2^, 33 cases (34.7%) had BMI between 18.50 - 23.99 kg/m^2^, 36 cases (37.9%) had BMI between 24.00 - 29.99 kg/m^2^ and 24 cases (25.3%) had BMI above 30.00 kg/m^2^ in the study group. The distribution of BMI of cases studied differs significantly between a group of cases with microalbuminuria and a group without microalbuminuria (P-value<0.05).

Of 95 cases studied with microalbuminuria, 72 cases (75.8%) were on oral antihyperglycemic agents (OHAs), 10 cases (10.5%) were on insulin, and 13 cases (3.7%) were on OHAs + insulin in the study group. The distribution of oral antidiabetic treatment received did not differ significantly between the group of cases with microalbuminuria and those without microalbuminuria (P-value 0.007).

Of 95 cases studied with microalbuminuria, 32 cases (33.7%) had fasting glucose below 126 mg/dl and 63 cases (66.3%) fasting glucose more than or equal to 126 mg/dl in the study group. Table 1 shows that the distribution of the fasting glucose level among the cases studied differs significantly between the group of cases with microalbuminuria and those without microalbuminuria (P-value 0.038)

**Table 1:** Inter-group comparison of fasting blood glucose among the cases studied Values are n and % of cases, P-value by the Chi-Square test. P-value<0.05 is considered to be statistically significant.  *P-value<0.05.

Parameter	Group A (n=95) (Microalbuminuria)		Group B (n=180) (Non-microalbuminuria)		P-value
Fasting glucose	n	%	n	%	0.038*
<126 mg/dl	32	33.7	84	46.7	
≥126 mg/dl	63	66.3	96	53.3	
Total	95	100.0	180	100.0	

Of 95 cases studied with microalbuminuria, 28 cases (29.5%) had post-prandial (PP) glucose below 200 mg/dl, and 67 cases (70.5%) had PP glucose more than or equal to 200 mg/dl in the study group. The distribution of the level of postprandial blood glucose among the cases studied did not differ significantly between the group of cases with microalbuminuria and the group of cases without microalbuminuria (P-value 0.457).

Of 95 cases studied with microalbuminuria, 32 (33.7%) had HbA1C levels below 6.5%, and 63 (66.3%) had HbA1C levels more than or equal to 6.5 % in the study group. Table 2 shows that the distribution of the level of HbA1C among the cases studied differs significantly between the group of cases with microalbuminuria and the group of cases without microalbuminuria (P-value>0.05).

**Table 2:** Inter-group comparison of HbA1C among the cases studied Values are n and % of cases, P-value by the Chi-Square test. P-value<0.05 is considered to be statistically significant.  *P-value<0.05.

Parameter	Group A (n=95) (Microalbuminuria)		Group B (n=180) (Non-microalbuminuria)		P-value
HbA1C	n	%	n	%	0.031*
<6.5 %	32	33.7	85	47.2	
≥6.5 %	63	66.3	95	52.8	
Total	95	100.0	180	100.0	

Of 95 cases studied with microalbuminuria, 84 cases (88.4%) had serum creatinine below 300 mg/g, and 11 cases (11.6%) had serum creatinine levels more than or equal to >300 mg/g in the study group. The distribution of the level of serum creatinine among the cases studied did not differ significantly between the group of cases with microalbuminuria and those without microalbuminuria (P-value>0.05).

Of 95 cases with microalbuminuria, 14 cases (14.7%) had a duration of diabetes between 1 and 4 years, 55 cases (57.9%) had a duration between 5 and 10 years, and 26 cases (27.4%) had a duration of diabetes more than 10 years. Table 3 shows that the distribution of duration of diabetes differs significantly between the group of cases with microalbuminuria and those without microalbuminuria (P-value<0.05).

**Table 3:** Inter-group comparison of the duration of diabetes Values are n and % of cases, P-value by the Chi-Square test. P-value<0.05 is considered to be statistically significant.  **P-value<0.05.

Parameter	Group A (n=95) (Microalbuminuria)		Group B (n=180) (Non-microalbuminuria)		P-value
Duration (years)	n	%	n	%	0.004**
1 – 4 years	14	14.7	39	21.7	
5 – 10 years	55	57.9	120	66.7	
>10 years	26	27.4	21	11.7	
Total	95	100.0	180	100.0	

Of 95 cases studied with microalbuminuria, 43 cases (45.3%) had raised QTc levels, and 52 (54.7%) did not have raised QTc levels in the study group. Table 4 shows that the distribution of incidence of raised QTc is significantly higher in the group of cases with microalbuminuria compared to the group of cases without microalbuminuria (P-value<0.05).

**Table 4:** Inter-group comparison of the incidence of raised QTc among the cases studied Values are n and % of cases, P-value by the Chi-Square test. P-value<0.05 is considered to be statistically significant. ***P-value<0.001. QTc: QT corrected

Parameter	Group A (n=95) (Microalbuminuria)		Group B (n=180) (Non-microalbuminuria)		P-value
Raised QTc	n	%	n	%	0.001***
Yes	43	45.3	18	10.0	
No	52	54.7	162	90.0	
Total	95	100.0	180	100.0	

Table 5 shows the mean corrected QT interval distribution according to age, sex, duration of diabetes, and the type of anti-diabetic treatment. The mean corrected QT interval distribution did not differ significantly across various age groups of cases studied with microalbuminuria (P-value>0.05). The distribution of mean corrected QT interval did not differ significantly between the group of male cases and group of female cases studied with microalbuminuria (P-value>0.05). The mean corrected QT interval distribution did not differ significantly across various duration of diabetes groups among the cases studied with microalbuminuria (P-value>0.05). The mean corrected QT interval distribution did not differ significantly across different types of anti-diabetic treatment groups among the cases studied with microalbuminuria (P-value>0.05).

**Table 5:** Distribution of mean corrected QT interval according to age, sex, duration of diabetes, and the type of anti-diabetic treatment among the cases studied OHA: Oral antihyperglycemic agent

Parameters		Corrected QT interval (ms)		
		No. of cases (n)	Mean	SD
Age group (years)	30 – 39	16	432.47	54.97
	40 – 49	17	437.09	46.56
	50 – 59	27	432.98	57.37
	60 – 65	35	431.36	43.98
	P-value		0.985	
Sex	Male	51	435.08	50.62
	Female	44	430.66	48.98
	P-value		0.668	
Duration of diabetes (years)	<5	27	436.59	49.22
	5 – 10	44	435.68	52.09
	>10	24	424.17	46.42
	P-value		0.603	
Type of treatment	OHA	72	435.74	49.26
	Insulin	10	422.80	60.48
	OHA + Insulin	13	425.92	45.04
	P-value		0.641	

## Discussion

CAN is a frequent and life-threatening complication of type 2 diabetes. Failure to diagnose can lead to high mortality and morbidity. A previous study done by Al-Shaikh in 2007 reported prevalence of microalbuminuria in diabetic patients was 45.6% and 39.8%, respectively [16]. In our study conducted at Bharati Hospital and Research Center, Pune, we found the association of corrected QT interval with microalbuminuria (CAN) in type 2 diabetes patients.

In the present study, the mean age of type 2 diabetes patients with microalbuminuria was 52.6 ± 10.2 years with a range of 30 - 65 years. Most type 2 diabetes with microalbuminuria patients (n=35) were 60-65 years (36.8%). 28.4% of patients were from 50-59 years, followed by the age group 40-49 years (17.9%) and the age group 30-39 years (16.8%). There was a significant difference in the distribution of age groups between type 2 diabetes patients with microalbuminuria and without microalbuminuria (P-value <0.001). In the study done by Oluk in 2019, the age of patients was from 33 to 81 years with an average of 57.52 ± 10.47 years which was significantly higher than that in our study [17].

In the present study, most type 2 diabetes patients with microalbuminuria were males (53.7%) compared to females (46.3%). The male-to-female gender ratio was 1.16:1. There was a significant prevalence of microalbuminuria in males than in females. There was a significant difference in gender distribution among the patients with microalbuminuria and those without microalbuminuria (P-value 0.046).

In a similar study by Sakthi et al. in 2021, the male-to-female ratio was 1.15:1, slightly lower than that in the present study [18]. Another study by Mehta et al. in 2002 reported a male-female ratio of 1.38:1 [19]. The study by Li in 2012 reported high prevalence in females than males, which is different from our current study [20].

In our present study, most type 2 diabetes patients with microalbuminuria (n=95, 37.9 %) had BMI between 24.00 and 29.99 kg/m^2^. 34.7% of patients had BMI between 18.50 and 23.99 kg/m^2^, followed by BMI less than 18.50 kg/m^2^ and BMI ≥ 30.00 kg/m^2^, which accounted for 2.1% and 25.3 %, respectively. In our study, there was a significant difference in the distribution of BMI between type 2 diabetes with microalbuminuria patients and without microalbuminuria (P-value 0.07). The previous study by Rutter in 2002 found a significant association of body mass index between type 2 diabetic patients with microalbuminuria and without microalbuminuria (Pvalue <0.01) [21]. In the present study, 57.9% of patients had diabetes for 5-10 years, compared to 27.4 % of patients who have had diabetes for more than 10 years. 14.7% of patients have had diabetes for 1-4 years. There was a significant difference in the duration of diabetes between patients with microalbuminuria and without microalbuminuria (P-value <0.05) (Table 3).

In the present study, most of the diabetic patients with microalbuminuria, i.e., 75.8%, were on OHAs. Subsequently, 10.5% of patients were on insulin, and 3.7% were on OHAs and insulin combination therapy. The difference in the distribution of type of treatment was not significant between type 2 diabetic patients with microalbuminuria and without microalbuminuria (P-value>0.05). The study done by Kazumi et al. reported a significant association between insulin treatment and an increase in corrected QT interval [22]. A similar finding was reported by Takebayashi et al. [23].

In the present study, the majority of diabetic patients with microalbuminuria (66.3%) had fasting glucose more than or equal to 126 mg/dl and 33.7% of diabetic patients had glucose below 126 mg/dl. There was a significant difference in raised fasting blood glucose levels between diabetic patients with microalbuminuria and without microalbuminuria (P-value < 0.05) (Table 1). In the present study, most diabetic patients with microalbuminuria (70.5 %), had more than 200 mg/dl PP glucose. 29.5% of patients had PP glucose levels below 200 mg/dl. There was no significant difference in PP levels between diabetic patients with microalbuminuria and without microalbuminuria (P-value>0.05). In the study by Li in 2012, they reported that patients with microalbuminuria had poor blood glucose control and reported postprandial glucose level as an independent risk factor for prolonged corrected QT interval (P-value <0.001) [20]. In the present study, the majority of diabetic patients with microalbuminuria (66.3 %) had HbA1C levels more than or equal to 6.5 % and 33.7 % of patients had HbA1C levels below 6.5%. There was a significant difference in HbA1C levels between diabetic patients with microalbuminuria and without microalbuminuria (P-value < 0.05) (Table 2). The study by Xian Li in 2012 also reported a significant increase in HbA1c levels in diabetic patients with microalbuminuria than in patients without microalbuminuria, which is 8.1 ± 2.1 and 7.6 ± 1.8, respectively, (P-value <0.01) [20].

In the present study, most diabetic patients with microalbuminuria (88.4 %) had Serum creatinine below 1.3 mg/dl. However, 11.6% of patients had more than or equal to 1.3 mg/dl serum creatinine levels. There was no significant difference in serum creatinine levels between diabetic patients with microalbuminuria and without microalbuminuria (P-value>0.05).

In the present study, most diabetic patients with microalbuminuria (54.7 %) did not have a prolonged corrected QT interval. 45.3 % of patients had prolonged corrected QT intervals. There was a significant difference in the corrected QT interval prolongation prevalence between diabetic patients with microalbuminuria and without microalbuminuria (P-value<0.05) (Table 4). In the present study, there was no significant association seen in the mean corrected QT interval according across various age groups (P-value 0.985), male and female patients (P-value 0.668), various duration of diabetes (P-value 0.603), and different types of anti-diabetic treatment (P-value 0.641). (Table 5). A similar finding was found in previous studies. For example, the study done by Zieler et al. in 1992 and another by Mathur et al. in 2006 reported corrected QT interval prolongation in 38.8% and 38%, respectively, similar to our current study findings [24,25]. A similar finding was found in another study by Rutter et al. in 2002. They reported that 67% of type 2 diabetic patients had prolonged corrected QT intervals, which was statistically significant [21]. A similar finding was found in a study by Basu et al. in 2010, which reported a significant association between type 2 diabetes patients with microalbuminuria and corrected QT interval prolongation (77.77%) [26].

The study by Li in 2012 reported a significant association between microalbuminuria and prolonged corrected QT interval prolongation [20]. Another study by Yeo et al. in 2004 reported a significant increase in prolonged corrected QT interval in type 2 diabetes patients with microalbuminuria than in patients without microalbuminuria [27]. The abovementioned literature supports the association of corrected QT interval prolongation with microalbuminuria in type 2 diabetes patients. However, different findings were reported in a study by Mustafa in 2021 and have not found a significant association between the corrected QT interval and diabetic patients with microalbuminuria [28].

This study has several limitations. First, this study has a potential selection bias compared to other population studies. Second, patients from only one hospital were included in this study which can cause a limit to this study. Third, in the present study, we measured the QT interval and not the other QT parameters, such as QT dispersion.

## Conclusions

This single-centered current study reported a significant association between microalbuminuria and prolonged QT interval. Findings of this study have both clinical and epidemiological significance. The early management of type 2 diabetes beginning from the early stages of the disease can reduce the risk of CAN, specifically for the Indian/Asian population where type 2 diabetes is prevalent. Therefore, these patients should be diagnosed as early as possible and treated to reduce associated mortality and risk.
